# Memory and Algorithmic Constraints of GPUs in Diffraction Data Processing

**DOI:** 10.1063/4.0001047

**Published:** 2025-10-27

**Authors:** Zbyszek Otwinowski, Raquel Bromberg

**Affiliations:** 1 UT Southwestern Medical Center, Dallas, TX, USA; 2 Ligo Analytics, Dallas, TX, USA

## Abstract

GPUs offer significant potential for accelerating diffraction data processing leading to potential speed-ups of up to 10,000-fold. However, achieving this potential is substantially limited by the extensive effort required to redesign algorithms so that they are optimized for GPU architectures. Diffraction data analysis inherently lends itself to vectorization, yet existing data structures frequently introduce serial dependencies incompatible with the parallel architecture of GPUs, severely diminishing computational efficiency. Historically, diffraction data algorithms development was heavily influenced by memory constraints, as memory-efficient algorithms often performed better and were faster on CPUs. Transitioning to GPUs introduces distinct challenges, notably designing computations around two- or three-dimensional grids, wherein each grid point's calculation is handled independently by dedicated GPU threads. This grid-based parallelism enables GPUs to manage hundreds of thousands of simultaneous threads effectively.

I will describe these challenges and opportunities with specific examples of both legacy and newly developed GPU-oriented algorithms. These include (a) transforming detector-coordinate data into a reciprocal lattice aligned with crystallographic axes, analogous to the traditional XDS algorithm, and (b) using libraries that streamline computational code development, by integration of GPU-based FFTs with intuitive coordinate system representations. Such approaches will allow us to fully leverage GPU capabilities in diffraction data analysis.

